# Effect of *Bacillus subtilis* and Oregano Oil on Performance, Gut Microbiome, and Intestinal Morphology in Pullets

**DOI:** 10.3390/ani13162550

**Published:** 2023-08-08

**Authors:** Hee-Jin Kim, Hyun-Soo Kim, Yeon-Seo Yun, Hwan-Ku Kang

**Affiliations:** Poultry Research Institute, National Institute of Animal Science, Rural Development Administration, Pyeongchang 25342, Republic of Korea; khj0175@korea.kr (H.-J.K.); kindkims1@korea.kr (H.-S.K.); yys1313@korea.kr (Y.-S.Y.)

**Keywords:** gut microbiome, pullet, *Bacillus subtilis*, oregano essential oil, layer hen

## Abstract

**Simple Summary:**

Supplementation with additives, such as *Bacillus subtilis* and oregano oil, has a positive impact on the poultry health and performance by promoting the development and diversity of cecal microbial communities. Here, we observed changes in feed conversion ratio, gut morphology, and cecal microbial composition when we had a basal diet supplemented that *Bacillus subtilis* and oregano oil in pullets. The results showed that *Bacillus subtilis* and oregano oil had no effect on weight change, but they had a positive effect on gut morphology. They also had a positive effect on the microbial changes in the cecum.

**Abstract:**

This study aimed to examine the effect of probiotics containing *Bacillus subtilis* and oregano essential oil on the growth performance, intestinal morphology, and cecal microbial composition in pullets aged 0–18 weeks. A total of 324 day-old Hy-Line Brown chicks were randomly assigned to three treatment groups, with six replicates per group and 18 birds per pen. The experimental treatments consisted of the following: a control group receiving a basal diet (Con), a group receiving a basal diet supplemented with 1 g/kg (3 × 10^8^ cfu/kg) of *Bacillus subtilis* (BS), and a group receiving a basal diet supplemented with 0.3 g/kg of oregano essential oil (ORO). The groups supplemented with BS and ORO demonstrated significantly higher villus height/crypt depth ratios than the Con group. Microbial richness was significantly higher in groups supplemented with BS (*p* = 0.0317) and ORO (*p* = 0.00794) than in the Con group. These findings revealed a distinct separation between gut microbial communities of the Con group and those supplemented with ORO, based on unweighted and weighted UniFrac indices. Therefore, supplementation with *Bacillus subtilis* and oregano oil improved the composition of the microbiota, suggesting their positive effects on the gut health of pullets.

## 1. Introduction

Cecal microbiota and bacterial fermentation play vital roles in enhancing nutrient absorption, eliminating harmful substances, preventing pathogen colonization, and regulating the overall health and performance of chickens [1]. These factors are crucial to bolster pathogen resistance and promote optimal chicken health and productivity [2]. Several scientific studies have provided evidence that the gut microbiota plays a significant role in regulating various aspects of host physiology [3]. These factors include feed-conversion efficiency, glucose metabolism, immune function, bone formation, and host growth [3]. The composition of intestinal microbiota in chickens is influenced by various factors, such as the physiological, immunological, and nutritional status of the host animals. Factors such as developmental stage, genotype, sex, housing environment, and nutritional supplementation have been reported to regulate the intestinal microbiota in chickens [3].

Probiotics have numerous benefits, such as stabilizing the intestinal microbiome, boosting the immune system, preventing pathogen colonization, reducing inflammation, decreasing ammonia levels, increasing volatile fatty acid production, and enhancing vitamin B synthesis [4]. Fijan [5] conducted a review that highlighted various species and strains of bacteria/yeast commonly utilized as probiotics. These include *Lactobacillus*, *Bifidobacterium*, *Saccharomyces*, *Enterococcus*, *Streptococcus*, *Pediococcus*, *Leuconostoc*, and *Bacillus*. A significant benefit of probiotics is their ability to reduce the colonization of potentially pathogenic bacteria in the gastrointestinal tract. This can lower the risk of economic losses on farms owing to mortality and reduce the chance of consumers encountering pathogenic bacteria in end-products [4]. In a previous study by Li et al. [6], simultaneous supplementation with an antibiotic growth promoter and *Bacillus subtilis* DSM17299 in the first 3 weeks of chicken growth increased the abundance of beneficial microbiota. This supplementation also improved intestinal morphology during the subsequent 6–16 weeks. Neijat et al. [7] demonstrated that *Bacillus subtilis* DSM29784 supplementation selectively enriched the beneficial bacteria in the cecal microbiota during the growth and development phases of chickens. This modulation of the microbiota results in improved growth and performance of chickens. Therefore, *Bacillus subtilis* offer the advantage of decreasing colonization by harmful bacteria in the gastrointestinal tract. This reduces the risk of economic losses from livestock mortality and lowers the chances of consumers being exposed to pathogenic bacteria in the end products.

Phytochemicals in plants have demonstrated growth-enhancing, antimicrobial, antioxidant, anti-inflammatory, and immunostimulatory properties by reducing the bacterial load and modulating gene expression associated with antioxidants and immunity [8]. These beneficial effects are attributed to the presence of bioactive compounds, including essential oils (EOs). Interestingly, plant-derived natural products such as EOs and nonvolatile plant extracts have the potential to be utilized as feed additives in poultry nutrition. These additives offer beneficial properties in terms of improving growth performance, enhancing nutrient utilization, and reducing the presence of pathogenic microbes [9]. Carvacrol, eugenol, and trans-cinnamaldehyde, derived from oregano, clove, and cinnamon essential oils, are potent bioactive compounds known for their beneficial properties. These widely used feed additives exhibit antimicrobial, immunostimulatory, anti-inflammatory, antioxidant, and growth-promoting properties. They also enhance gut barrier integrity, improve digestibility, and modulate the intestinal microbiota [8].

Previous studies have highlighted the significance of conducting thorough analyses of gut microbiota across various developmental stages. Such comprehensive studies could provide valuable insights into the selection and appropriate use of probiotics at specific physiological stages. However, knowledge regarding egg type chicks is limited, particularly during the rearing or development of the pullet phase. Most studies on *Bacillus subtilis* and oregano oil have been conducted in broilers and have shown promising results. However, there is a scarcity of studies exploring their use in pullets and laying hens. Hence, this study aimed to investigate the effect of supplementing pullets with probiotics and oregano oil on their performance and to assess the effects of potentially pathogenic bacteria on the gut microbiome.

## 2. Materials and Methods

The experimental protocol was reviewed and approved by the Institutional Animal Care and Welfare Committee of the National Institute of Animal Science, Rural Development Administration of the Republic of Korea (2021-508).

### 2.1. Animals and Experimental Design

A total of 324 day-old Hy-Line Brown chicks were randomly divided into three groups, each with six replicates (18 birds per cage, 75 × 60 × 40 cm, L × W × H) in a closed conventional shed. The three groups were maintained at equal stocking densities of 0.025 m^2^/birds. Experimental groups included the following: a basal diet as control (Con), basal diet plus 3 × 10^8^ cfu/kg feed *Bacillus subtilis* (BS), and basal diet plus 0.3 g/kg feed oregano essential oil (ORO). The control diet was based on corn and soybean meal. The chicks were fed a control starter diet containing 18.5% crude protein, 3.7% crude fat, 1.1% calcium, 0.64% phosphorus, and 2,850 kcal/kg calculated metabolizable energy from 0 to 5 weeks; a grower diet containing 16.5% crude protein, 3.1% crude fat, 1.0% calcium, 0.6% phosphorus, and 2820 kcal/kg calculated metabolizable energy from 6 to 12 weeks; and then a pre-lay diet with 15.0% crude protein, 3.8% crude fat, 1.8% calcium, 0.53% phosphorus, and 2800 kcal/kg calculated metabolizable energy from 13 to 18 weeks. Additive *Bacillus subtilis* (Envia Pro 201 GT) was provided by Danisco USA Inc. (New Century, KS, USA), and oregano oil (Orego-Stim) was provided by Anpario Plc. (Nottinghamshire, UK). The experiment was conducted in pullets between 0 and 18 weeks of age. On the first day, the temperature was set at 34 °C, and it gradually decreased by 2 °C per week until it stabilized at 21 °C. The lighting program started off on the first day at 50 lux (22 light:2 dark). The lighting management was customized by using management guidelines for Hy-Line Brown laying hens throughout 0–18 week. Feed and water were provided ad libitum throughout the experimental period.

### 2.2. Growth Performance

All chicks were weighed at weeks 0, 6, 11, and 18. Feed intake of pullets was measured at weeks 6, 11, and 18; feed conversion ratio (FCR) was calculated as the ratio of feed intake to the gained body weight.

### 2.3. Analyses of HE:LY Ratio in Blood

Blood was collected from the wing vein at 18 weeks of age in EDTA-treated Becton Vacutainer tubes (Becton Dickinson, Franklin Lakes, NJ, USA). Whole blood samples from the pullets were immediately analyzed for heterophils (HE) and lymphocytes (LY) using the Hemavet Multi-Species Hematology System (Drew Scientific Inc., Oxford, CT, USA).

### 2.4. Ileal Morphology

At the end of the feeding trial, one bird per pen, with a body weight close to the average, was chosen for euthanasia using CO_2_ asphyxiation. Following euthanasia, the carotid artery was cut to exsanguinate the chick. Then, 3 cm long sections from the distal ileal (the one-third section from the ileal-cecal-colic junction to the Meckel’s diverticulum) were collected. The ileal of the pullet was fixed using 4% paraformaldehyde, dehydrated with a gradient of ethanol, completely encapsulated in paraffin, and sliced at a thickness of 5 μm made using a rotary microtome. Afterwards, sections were stained with hematoxylin and eosin (H&E). Images were obtained using a microscope (Nikon DS-Ri2; Nikon, Tokyo, Japan), and histomorphometric evaluations were performed using imaging software (NIS-Elements BR 4.0; Nikon, Tokyo, Japan).

### 2.5. 16S rRNA Analysis of Bacterial Cecal Composition

Fresh cecal content from two whole cecal pouches was aseptically collected, pooled, and immediately stored at −80 °C. DNA extraction was performed using POWERSoil DNA kit (Qiagen, Hilden, Germany) following the manufacturer’s instructions. The hypervariable region (V3–V4) of bacterial 16S rDNA was amplified using a fusion primer. Quality scores for the sequencing reads, such as base sequence quality, quality score, GC content, and N content, were checked using FastQC (v0.10.1). DADA2 method was used to remove low-quality reads, phiX reads, chimeric reads, and duplicate reads, and to infer amplicon sequence variants. Raw reads were uploaded to Quantitative Insights into Microbial Ecology 2 (QIIME2) and qualitatively trimmed. The 16S rRNA Silva database, which contains taxonomic information on bacteria, was used to assign taxonomies. Krona charts were generated showing the relative abundance of each sample according to phylogenetic order. Analysis of the composition of microbiomes with Bias Correction (ANCOM-BC) was used to assume differential taxa from each group.

### 2.6. Statistical Analyses

All data were analyzed via one-way analysis of variance using SAS software (version 9.4; SAS Institute Inc., Cary, NC, USA) to determine effects of BS and ORO. All statistical analyses were performed with individual chicks as the experimental unit, and analyses of feed intake and FCR were performed with a pen as experimental unit. Statistical differences among groups were determined using Tukey’s multiple range test. Differences were considered statistically significant at *p* < 0.05.

## 3. Results

### 3.1. Growth Performance

Table 1 shows the effects of dietary supplementation with BS and ORO on pullet production. Growth performance indices (body weight, body weight gain, feed intake, and feed conversion ratio) differed among the groups, but not significantly (*p* > 0.05).

### 3.2. HE:LY Ratio

The HE:LY ratio is a proven stress marker in chickens [10]. HE was significantly lower in the BS groups than in the Con and ORO group. No significant difference was found in LY in the blood. As shown in Table 2, BS and ORO group showed a significant (*p* < 0.05) decrease in the HE/LY ratio.

### 3.3. Histomorphological Analysis

**Figure 1:**
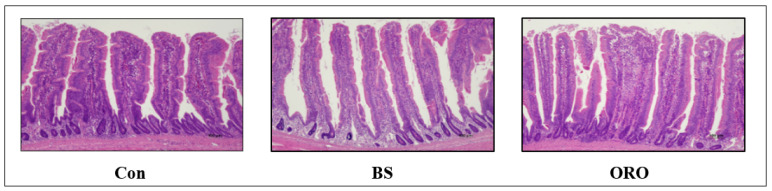
Dietary *Bacillus subtilis* and oregano oil supplementation on intestinal morphology of pullet. Con: control group receiving a basal diet; BS: basal diet supplemented with 1 g/kg (3 × 10^8^ cfu/kg) of *Bacillus subtilis*; ORO: basal diet supplemented with 0.3 g/kg of oregano essential oil.

The mean values of villus height, crypt depth, and villus height/crypt depth ratio from the distal ileal samples are shown in Table 3. Villus height significantly increased in pullets fed BS. Crypt depths were significantly lower in the BS and ORO groups than in the Con group. Therefore, BS and ORO groups showed higher villus height/crypt depth ratios than the control group. Also, the results in Figure 1 show that BS and ORO treatments have a longer villus and shallower crypts than Con.

### 3.4. Cecal Microbial Profile

**Figure 2:**
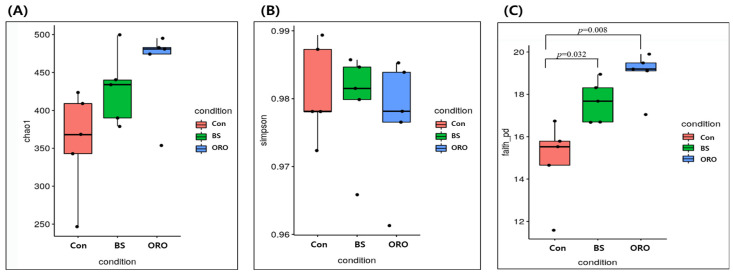
Effect of dietary *Bacillus subtilis* and oregano oil supplementation on the Chao1 (**A**), Simpson’s evenness (**B**), and Faith’s phylogenetic diversity (**C**) of cecal microbiota in pullets. Con: control group receiving a basal diet; BS: basal diet supplemented with 1 g/kg (3 × 10^8^ cfu/kg) of *Bacillus subtilis*; ORO: basal diet supplemented with 0.3 g/kg of oregano essential oil.

Alpha diversity indices (Chao1, Simpson, and Faith’s PD) were investigated and are shown in Figure 2. There were no significant differences (*p* > 0.05) between Chao1 and Simpson’s evenness values. Microbial richness (i.e., Faith’s PD) was significantly greater in BS (*p* = 0.0317; Figure 2C) and ORO (*p* = 0.00794) groups than in the control group.

**Figure 3:**
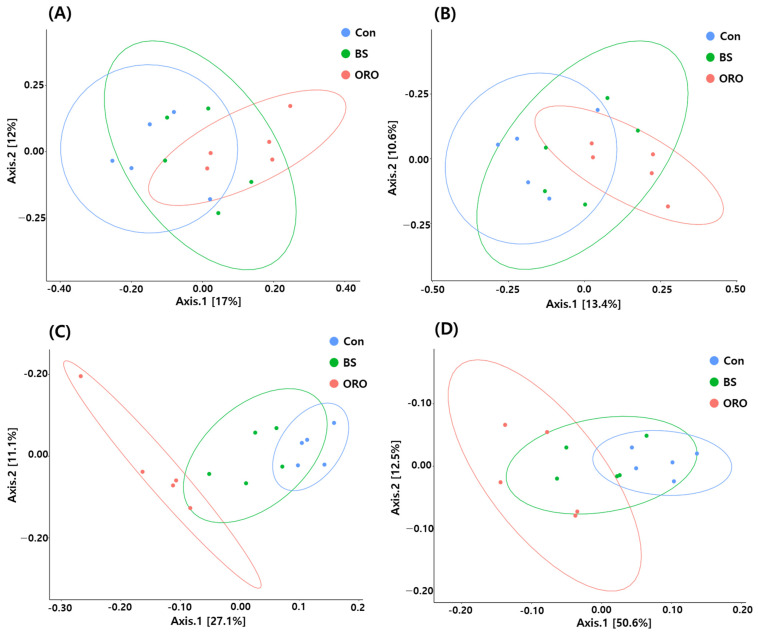
Relationship between bacterial communities sampled from pullets after supplementation with *Bacillus subtilis* and oregano oil for 18 weeks. (**A**): Bray–Curtis similarity; (**B**): Jaccard similarity, (**C**): unweighted UniFrac; (**D**): weighted UniFrac distances with 95% confidence ellipses. Con: control group receiving a basal diet; BS: basal diet supplemented with 1 g/kg (3 × 10^8^ cfu/kg) of *Bacillus subtilis*; ORO: basal diet supplemented with 0.3 g/kg of oregano essential oil.

The study employed a principal coordinate analysis (PCO) plot of the Bray–Curtis similarity, Jaccared similarity, unweighted UniFrac, and weighted UniFrac indices to investigate the structure of the gut microbiota (Figure 3). Feed additives were considered in the analysis. The results of the Bray–Curtis similarity and Jaccared similarity indices were evidenced by a similar cluster formation following different groups. This suggests that the feed additive had limited impact on the microbiota. However, the results showed a clear separation between gut ceca of Con and ORO groups in the unweighted UniFrac and weighted UniFrac indices.

**Figure 4:**
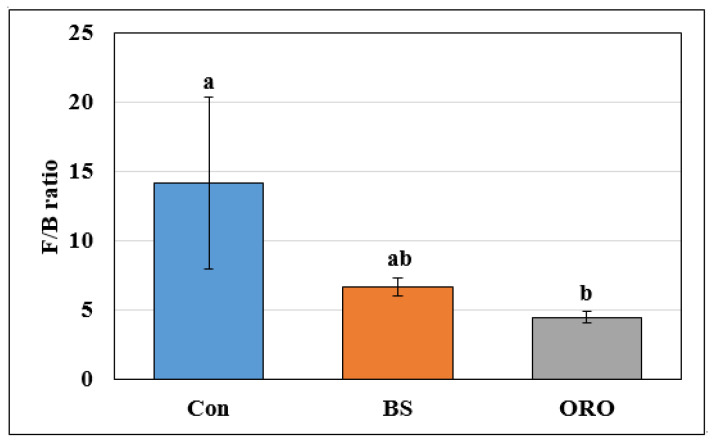
Composition of gut bacteria from pullets after supplementation with *Bacillus subtilis* and oregano oil for 18 weeks at Firmucutes/Bacteriodetes ratio (Phylum level). Data shown as the mean ± SE. One-way analysis of variance with Tukey’s post hoc test was used. Con: control group receiving a basal diet; BS: basal diet supplemented with 1 g/kg (3 × 10^8^ cfu/kg) of *Bacillus subtilis*; ORO: basal diet supplemented with 0.3 g/kg of oregano essential oil. ^a,b^ Means in the treatment with different superscripts are significantly different (*p* < 0.05).

Overall, at the phylum level, ORO supplementation increased the populations of Bacteroidetes and Proteobacteria mainly at the expense of Firmicutes (Figure 4).

**Figure 5:**
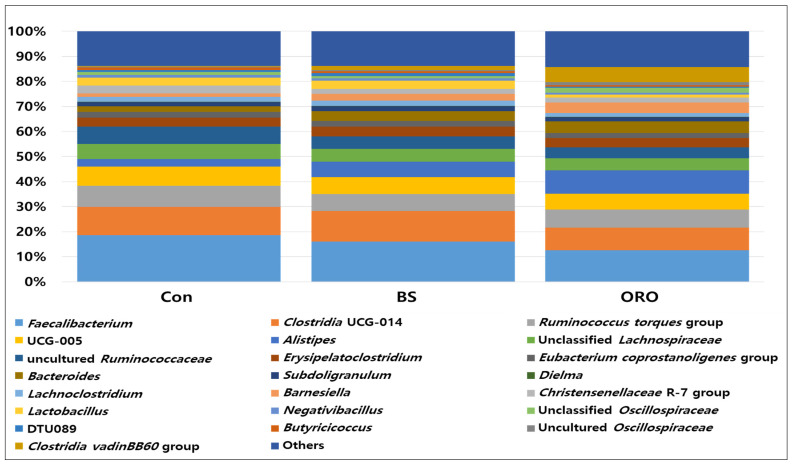
The composition of gut bacteria at genus from pullets after supplementation with *Bacillus subtilis* and oregano oil for 18 weeks (*n* = 5/group). Con: control group receiving a basal diet; BS: basal diet supplemented with 1 g/kg (3 × 10^8^ cfu/kg) of *Bacillus subtilis*; ORO: basal diet supplemented with 0.3 g/kg of oregano essential oil.

The most dominant microbiota at genus levels is shown in Figure 5. The most abundant and prevalent genera observed in the Con group included *Faecalibacterium* (18.7%) followed by *Clostridia* UCG-014 (11.3%), the *Ruminococcus torques* group (8.4%), UCG-005 (7.6%), and uncultured *Ruminococcaceae* (6.8%). In the BS group, the most common bacterial found were *Faecalibacterium* (15.9%), *Clostridia* UCG-014 (12.2%), *Ruminococcus torques* group (6.8%), UCG-005 (6.7%), and *Alistipes* (6.2%). However, in the ORO group, the most common bacteria found were *Faecalibacterium* (12.6%), *Alistipes* (9.4%), *Clostridia* UCG-014 (8.9%), the *Ruminococcus torques* group (7.2%), and *Clostridia* UCG-014 (6.2%). *Alistipes* were 3.02, 6.21, and 9.39% in the Con, BS, and ORO groups, respectively. In the Con group, *Bacteroid*es, known for their involvement in the breakdown of isoflavone genistein, accounted for 2.16% of the microbiota. However, in the BS and ORO groups, *Bacteroides* represented 4.03% and 4.61%, respectively.

**Figure 6:**
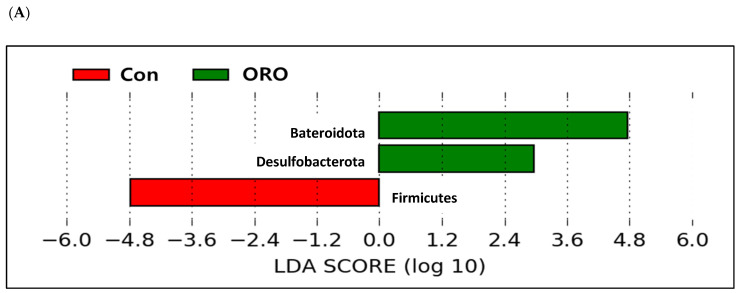

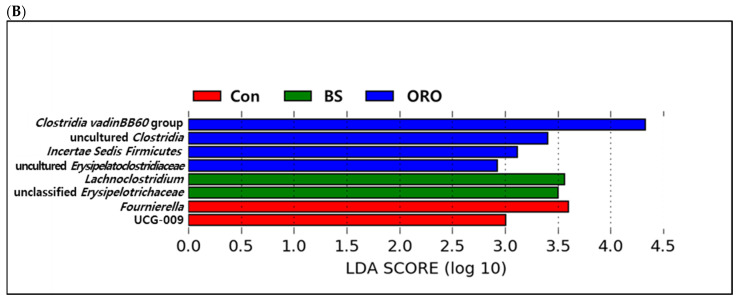
Comparison of microbial communities between supplementation with *Bacillus subtilis* and oregano oil using linear discriminant analysis (LDA) combined with effect size measurements (LEfSe) at phylum (**A**) and genus (**B**) levels in pullets. Con: control group receiving a basal diet; BS: basal diet supplemented with 1 g/kg (3 × 10^8^ cfu/kg) of *Bacillus subtilis*; ORO: basal diet supplemented with 0.3 g/kg of oregano essential oil.

An LDA analysis of Con, BS, and ORO groups revealed significant differences in three (Figure 6A) and eight genera (Figure 6B). The Con group had a higher abundance of phyla Firmicutes than the ORO group, while the phylum Bacteroidetes and Desulfobacterota were enriched in the ORO group (*p* < 0.05; Figure 6A). Genera *Clostridia* vadinBB60 group, uncultured *Clostridia*, *Incertae Sedis Firmicutes*, and uncultured *Erysipelotrichaceae* were enriched in the ORO group (*p* < 0.05; Figure 6B). The genera *Lachnoclostridium* and unclassified *Erysipelotrichaceae* were enriched in the BS group (*p* < 0.05, Figure 6B). The Con group was enriched in the genera *Fournierella* and UCG-009 (*p* < 0.05; Figure 6B).

## 4. Discussion

Owing to the limitations of the use of antibiotics as growth promoters for animals, there has been growing interest in the use of natural bioactive compounds, such as EOs (oregano oil), to enhance poultry health and performance [11]. Positive effects observed on feed utilization may be attributed to the presence of active components, specifically thymol and carvacrol, in oregano oil [11,12]. These components have been shown to have antimicrobial and anti-inflammatory properties and may contribute to enhancing gut health status and nutrient utilization [13]. *Bacillus subtilis* secretes potent proteases, lipases, and amylases that break down complex carbohydrates in plants, thus increasing the digestibility of nutrients and providing animals with more nutrition [14]. According to Zhang et al. [15], B. subtilis enhances the growth performance of chickens. Dietary *Bacillus subtilis* at 200 mg/kg significantly improved the growth performance of broilers [16], and *Bacillus subtilis* supplementation improved FCR in broilers infected with *Salmonella* [17]. Brenes and Roura [18] and Hippensteil et al. [19] observed that incorporating EOs from certain herbs into diets at concentrations ranging from 12 to 300 ppm had positive effects on the performance; however, there was no consistent effect on feed intake.

Heterophils (HE) are one of the most abundant types of granulocytes in most avian species. They play crucial roles in the avian immune system, being responsible for phagocytosis and possessing bactericidal properties [20]. Moreover, HE are essential in mediating acute inflammation, playing a significant role in the bird’s defense against pathogens and infections [20]. In birds, HE numbers tend to rise during periods of mild to moderate stress. The HE:LY ratio can be utilized as a reliable method to identify the presence of physiological stress in birds [21]. In the current study, BS and ORO in pullets diets lowered the HE:LY ratio. Tang et al. [22] reported that the inclusion of probiotics (*Lactobacillus acidophilus*, *Lactobacillus casei*, *Bifidobacterium bifidum*, and *Streptococcus faecium*) in hen diets decreased the HE:LY ratio. Wang et al. [23] found that HE:LY ratios in *B. subtilis*-fed broiler were lower than in Control. Yesilbag et al. [24] reported that HE:LY ratio was determined to have differences in the oregano and rosemary oil supplementation groups compared with the control group in Pharaoh quails. Therefore, it seems that the feeding of BS and ORO reduced the HE:LY ratio in the blood, which reduced the stress of the laying hens.

BS and ORO feeds induced changes in the histomorphology of the ileum; a significant increase in the villus height/crypt depth ratio was observed (Table 3). The small intestine is an important site for digestion and absorption of nutrients, and absorption efficiency is particularly dependent on the histomorphology of the small intestine [25]. One key feature indicating a healthy digestive tract in birds is the presence of an elongated and intact villus along with shallow crypts [26]. Increased villus length improves nutrient absorption capacity and increases the mucosal surface area [27]. Additionally, when estimating the digestive capacity of the small intestine, the villus height/crypt depth ratio is considered standard [28]. Numerous studies have documented the positive effects of probiotic (*B. amyloliquefaciens* and *Bacillus subtilis*) supplementation on the intestinal ultrastructure of broilers [29,30]. Although the significance of the cecum in well-being, productivity, and illness of chickens is widely acknowledged, limited studies have been conducted on the microbiota of chicken cecum [31]. Gut microbiota plays a crucial role in the gastrointestinal system of animals significantly contributing to the digestive processes. Dietary interactions substantially influenced the composition of the intestinal microbial community [32]. Dietary probiotics positively affect the gut microbiome [32]. Supplementation with probiotics can promote the proliferation of beneficial bacteria, while reducing the population of potentially harmful bacteria in the intestinal tract of chickens. Furthermore, it aids in restoring the intestinal microbial dysfunction caused by pathogens [29].

The Con group was significantly different from the BS and ORO groups (Faith’s phylogenetic diversity). The addition of BS and ORO significantly changed the community composition and relative abundance of specific microorganisms. The variation in cecum microbial populations could be attributed to the diverse range of active ingredients present such as antioxidant and antibacterials in BS and ORO and the varying sensitivities of microorganisms [33]. The gut microbiota consists of a diverse and intricate combination of bacterial phyla, with Firmicutes and Bacteroidetes as the predominant phyla. Relative abundances of Bacteroidetes and Firmicutes in the gut microbiota can influence the host metabolism. In the ORO group, researchers observed a notable decrease in Firmicutes and an increase in Bacteroidetes within chicken intestinal microbiota. This results in a substantial reduction in the Firmicutes to Bacteroidetes (F/B) ratio. In studies in mice, pigs, and humans, the two major bacterial phyla, Firmicutes and Bacteroidetes, primarily correlated with increased BW [34]. However, no such correlations were observed in the present study. Bacteroidetes are bacteria that inhabit the gut. They help the body to break down polysaccharides, which are complex carbohydrates that are not easily digested. This helps improve nutrient absorption and boost the immune system. Bacteroidetes also help maintain the gut microbiota in balance, which is important for the overall health [35]. Guo et al. [36] reported that the gut microecosystems of Pine (*Pinus massoniana* Lamb.) Needle Extract-fed layer hens favored Bacteroidetes at expense of Firmicutes, thus leading to a lower Firmicutes/Bacteroidetes ratio. The addition of ORO significantly altered the community composition and relative abundance of certain microorganisms, which may have been due to the active ingredients in ORO and the sensitivity of various microorganisms to ORO.

*Alistipes* had higher values in BS and ORO than Con. According to Polansky et al. [37], *Alistipes* possesses enzymes responsible for propionate synthesis. Propionate is an important short-chain fatty acid playing a crucial role in the gut development by serving as a vital nutrient source [37]. The percentage of *Bacteroides* in the cecal microbiota was higher in the BS and ORO treatments than in the Con group. Gauffin et al. [38] found potential benefits of *Bacteroides* in improving the metabolic and immunological dysfunction in mice with HFD-induced obesity. This suggests that a higher abundance of *Bacteroide*s, associated with the degradation of genistein, could be advantageous for maintaining gut health. The combined presence of these dominant bacteria positively contributed to the development and growth of host pullets, indicating their beneficial effects. Both *Alistipes* and *Bacteroides* have been reported to exhibit positive correlations with an increased FCR [3]. This observation aligns with a previous study by Neijat et al. [7], which established a strong association between *Alistipes* and nutrient retention variables linked to the growth performance of pullets aged 5–10 weeks [7]. Khan and Chousalkar [39] also found that *Bacillus*-based probiotic supplementation enhanced the abundance levels of *Bacteroides* and *Alistipes* in cecal of layer hen. The cecal microbiota may thus play a role in the metabolic regulation and development of pullets at various growth stages. This implies that the addition of probiotics could potentially exert a favorable influence on the growth and health of pullets.

## 5. Conclusions

In conclusion, this study aimed to enhance pullet productivity and health by administering Bacillus subtilis and oregano oil. These interventions played a significant role in promoting animal welfare through reduced H/L ratio and increased villus length, enhancing nutrient absorption. Additionally, diets A and B induced alterations in the cecal microbiome, suggesting potential growth of beneficial bacteria and nutrient utilization improvement. The observed enhancements in pullet production and health underscore the practicality of continuous utilization of Bacillus subtilis and oregano oil. To sum up, incorporating these additives in pullet diets yielded favorable outcomes during their developmental phase.

## Figures and Tables

**Table 1:** Effect of dietary *Bacillus subtilis* and oregano oil supplementation on production performance of pullets.

		Con	BS	ORO	SEM ^1^	*p*-Values
Initial body weight		39.57	39.72	39.67	0.113	0.6247
0–6 weeks	Body weight	451.73	464.17	440.48	8.720	0.1997
	Body weight gain	412.16	424.45	400.81	8.682	0.1987
	Feed intake	694.57	735.69	707.30	27.210	0.5653
	FCR	1.68	1.74	1.76	0.047	0.5372
6–11 weeks	Body weight	995.43	989.21	970.48	15.499	0.5147
	Body weight gain	543.70	525.04	530.00	13.032	0.5909
	Feed intake	2352.39	2347.34	2398.27	32.366	0.4925
	FCR	4.33	4.47	4.54	0.094	0.3195
11–18 weeks	Body Weight	1524.11	1538.55	1507.17	19.202	0.5303
	Body weight gain	528.68	549.33	536.69	15.399	0.6436
	Feed intake	4665.72	4421.52	4424.55	112.507	0.2516
	FCR	8.84 ^a^	8.07 ^b^	8.24 ^b^	0.132	0.0036
Over all weeks	Body weight gain	1484.54	1498.82	1467.50	19.194	0.5310
	Feed intake	7712.68	7504.54	7530.12	112.597	0.3911
	FCR	5.20	5.01	5.13	0.060	0.1220

^1^ SEM, standard error of means; Con: control group receiving a basal diet; BS: basal diet supplemented with 1 g/kg (3 × 10^8^ cfu/kg) of *Bacillus subtilis*; ORO: basal diet supplemented with 0.3 g/kg of oregano essential oil; FCR: feed conversion ratio. ^a,b^ Means in the same row with different superscripts are significantly different (*p* < 0.05).

**Table 2:** Effect of dietary *Bacillus subtilis* and oregano oil supplementation on heterophil to lymphocyte ratio in pullets.

	Con	BS	ORO	SEM ^1^	*p*-Values
HE	8.17 ^a^	6.61 ^b^	7.43 ^a^	0.174	0.0007
LY	13.58	12.74	13.42	0.451	0.0913
HE:LY	0.60 ^a^	0.52 ^b^	0.55 ^b^	0.051	0.0002

^1^ SEM, standard error of means; Con: control group receiving a basal diet; BS: basal diet supplemented with 1 g/kg (3 × 10^8^ cfu/kg) of *Bacillus subtilis*; ORO: basal diet supplemented with 0.3 g/kg of oregano essential oil; HE: hematophil; LY: lymphocyte. ^a,b^ Means in the same row with different superscripts are significantly different (*p* < 0.05).

**Table 3:** Effect of dietary *Bacillus subtilis* and oregano oil supplementation on histomorphological parameters of the distal ileum section of pullets.

	Con	BS	ORO	SEM ^1^	*p*-Values
Villus height (µm)	663.29 ^b^	720.98 ^a^	679.28 ^b^	13.478	0.0094
Crypt depth (µm)	130.18 ^a^	120.00 ^b^	117.31 ^b^	3.338	0.0187
Villus height/Crypt depth	5.19 ^b^	6.12 ^a^	5.91 ^a^	0.164	0.0003

^1^ SEM, standard error of means; Con: control group receiving a basal diet; BS: basal diet supplemented with 1 g/kg (3 × 10^8^ cfu/kg) of *Bacillus subtilis*; ORO: basal diet supplemented with 0.3 g/kg of oregano essential oil. ^a,b^ Means in the same row with different superscripts are significantly different (*p* < 0.05).

## Data Availability

Not applicable.
